# Research on the Anti-Ultraviolet Aging Performance of Fishery HDPE/UHMWPE-Blended Monofilaments

**DOI:** 10.3390/polym18030392

**Published:** 2026-02-01

**Authors:** Zun Xue, Jiangao Shi, Jian Zhang, Wenyang Zhang, Dong Jin, Yihong Chen, Ying Ding, Hongzhan Song, Pei Han

**Affiliations:** 1 East China Sea Fisheries Research Institute, Chinese Academy of Fishery Sciences, Shanghai 200090, China; xuezun2023@163.com (Z.X.); wyzhang@ecsf.ac.cn (W.Z.); jindong1117@zjou.edu.cn (D.J.); 18658071579@163.com (Y.C.); dingying0901@163.com (Y.D.); song15163859308@163.com (H.S.); 17501646621@163.com (P.H.); 2 College of Marine Sciences, Fujian Agriculture and Forestry University, Fuzhou 350002, China; 3 College of Marine Biological Resources and Management, Shanghai Ocean University, Shanghai 201306, China; j-zhang@shou.edu.cn

**Keywords:** HDPE, UHMWPE, blended monofilaments, ultraviolet aging, fishing materials

## Abstract

To enhance the anti-ultraviolet aging capacity of high-density polyethylene (HDPE) monofilaments for fishery applications, this study prepared pure HDPE and a blend of HDPE/UHMWPE (80/20 wt%) monofilaments via a melt spinning process. Systematic ultraviolet accelerated-aging experiments were conducted on these monofilaments for durations ranging from 0 to 600 h. The evolution of material properties was assessed using various quantitative characterization methods, including scanning electron microscopy (SEM), Fourier transform infrared spectroscopy (FTIR), thermogravimetric analysis (TGA), differential scanning calorimetry (DSC), and mechanical tensile testing. The results indicate that after 600 h of aging, the density and size of surface cracks in the blended monofilament are significantly lower than those observed in pure HDPE. The carbonyl index (*CI*) and unsaturated index (*UI*) of the blend are approximately 55% and 40% of those of pure HDPE, respectively. Additionally, the initial thermal decomposition temperature (*T*_5%_), as determined by TGA, decreases by only 13 °C, which is a considerably lower reduction than the 28 °C observed for pure HDPE. Furthermore, the attenuation rates of breaking strength and elongation at break for the blended monofilament are 43.7% and 54.0%, respectively, which are markedly lower than the corresponding rates of 54.5% and 66.0% for pure HDPE. Research indicates that the observed performance improvement is closely linked to the synergistic mechanism of the “physical hindration–structural skeleton” formed by the UHMWPE phase. Furthermore, this mechanism may interact synergistically with the antioxidants present in the system, thereby altering the material’s failure mode from “rapid brittle failure” to “progressive slow deterioration.” This study offers novel modification strategies and experimental references for developing high-performance, UV-resistant polyolefin materials suitable for fishery applications.

## 1. Introduction

Marine fisheries play a crucial role in providing protein and supporting human livelihoods, while high-performance fishing materials are fundamental to their sustainable development [1]. Notably, fishing monofilaments composed of high-density polyethylene (HDPE) offer several advantages, including high specific strength, flexibility, excellent corrosion resistance, and relatively low costs for raw materials and processing. Consequently, they have emerged as essential materials for the production of marine aquaculture facilities, trawls, traps, various fishing nets, and different types of ropes [2,3,4,5,6]. The marine environment in which fishing materials are utilized is exceptionally harsh, necessitating prolonged exposure to a complex aging field characterized by factors such as high-intensity solar ultraviolet (UV) radiation [7]. Among these factors, solar ultraviolet radiation is considered the primary environmental agent responsible for inducing photo-oxidative aging in polymer materials, ultimately causing premature material failure [8,9]. When exposed to ultraviolet rays, polyethylene molecular chains can be directly excited, generating alkyl radicals that initiate a series of chain oxidation reactions. This process leads to molecular chain breakage, cross-linking, and the formation of oxygen-containing functional groups, including carbonyl and hydroxyl groups [10,11]. Macroscopically, this process gradually deteriorates the material surface, which becomes rough, powdery, discolored, and brittle. Concurrently, its mechanical properties, particularly elongation at break and impact toughness, exhibit a significant and irreversible decline. Ultimately, this deterioration results in insufficient strength and damage to the net equipment, leading to the escape of farmed organisms, economic losses, and environmental issues, including marine microplastic pollution [12].

In industrial applications, the aging process is typically mitigated by incorporating light stabilizers, such as hindered amine light stabilizers (HALSs), and ultraviolet absorbers. Nonetheless, these additives present challenges, including migration, exudation, dissolution in seawater, and potential environmental impacts [13]. Consequently, focusing on the polymer matrix and employing blending modification technology to develop new materials that exhibit both superior initial performance and long-term durability has emerged as a promising and valuable research avenue in the domain of fishery materials. Ultra-high-molecular-weight polyethylene (UHMWPE) possesses a molecular weight exceeding one million. Its highly entangled molecular chains and fully crystalline structure, among other structural characteristics, confer exceptional wear resistance, high impact toughness, excellent self-lubrication, and notable chemical corrosion resistance [14,15,16,17]. Blending UHMWPE with high-density polyethylene (HDPE) as the reinforcing phase creates a microstructure that combines rigidity and flexibility while preserving the processing advantages of HDPE, including ease of melt molding [18]. In this composite, HDPE functions as the continuous phase, providing the matrix and facilitating processability, while the dispersed UHMWPE phase serves as physical crosslinking points and stress-bearing frameworks. This arrangement enhances the material’s resistance to crack initiation and propagation.

Although the aforementioned blending strategies have demonstrated potential, and research has reported on the mechanical, friction, and conventional processing properties of HDPE/UHMWPE blends [19,20,21], a significant knowledge gap persists regarding the behavioral evolution and internal mechanisms of these blends under prolonged ultraviolet aging. Most current studies concentrate on blend systems with high UHMWPE content or depend on chemical additives to improve weather resistance. However, systematic experimental verification is lacking to determine whether low-content UHMWPE can achieve long-term UV aging resistance through purely physical mechanisms under standard melt processing conditions. Furthermore, the existing understanding primarily focuses on macro performance comparisons. There remains a deficiency in multi-scale and mechanics-based empirical explanations of how the entanglement network formed by the ultra-long molecular chains of UHMWPE evolves during the continuous and dynamic aging process and how it actively influences the overall failure path of the material, rather than merely delaying the decline of a single performance index.

This paper investigates HDPE/UHMWPE (80/20 wt%)-blended fishery monofilaments, which exhibit excellent processing feasibility. This study employs systematic ultraviolet accelerated aging experiments and multi-scale characterization to achieve several objectives. First, this study aims to comprehensively characterize and compare the multi-scale dynamic attenuation laws of pure HDPE and its blend system throughout the aging process. This characterization will encompass surface morphology, chemical structure, crystallization behavior, thermal stability, and macroscopic mechanical properties. Second, we will investigate how low-content UHMWPE contributes to a synergistic effect characterized by “physical hindrance” and “structural skeleton” through its unique ultra-long molecular chain. Finally, the findings will elucidate how the incorporation of UHMWPE is linked to a transformation in the material’s aging failure mode, shifting from “rapid brittle failure” to “progressive slow deterioration.” This study not only enhances the understanding of the photo-oxidative aging mechanisms in polyolefin blends but also offers new design concepts and a solid theoretical foundation for developing durable, processable, and environmentally friendly green fishery polyolefin materials.

## 2. Materials and Methods

### 2.1. Materials

Fishery high-density polyethylene (HDPE, grade 5000S), supplied by Sinopec Yangzi Petrochemical Co., Ltd. Nanjing, Jiangsu, China, exhibits a melt mass flow rate of 0.9 g/10 min at 190 °C under a load of 2.16 kg. This melt flow rate is conducive to melt spinning applications. Fishery ultra-high-molecular-weight polyethylene (UHMWPE, grade LL-1040), provided by Lianle Chemical Technology Co., Ltd. Shanghai, China, possesses a relative molecular mass of approximately 1.5 × 10^6^. Its exceptionally high molecular weight is critical for establishing a reinforced entanglement network. To enhance the compatibility of the two phases, polyethylene grafted with maleic anhydride (PE-g-MA, grade 4351) was incorporated as a compatibilizer, sourced from Clariant Chemical (China) Co., Ltd. Tianjin, China, Additionally, to mitigate thermal oxidation during processing, a composite antioxidant, consisting of primary antioxidant 1010 and auxiliary antioxidant 168 in a mass ratio of 1:1, was utilized and supplied by BASF AG.

In this study, we selected a blending ratio of HDPE/UHMWPE at 80/20 (wt%). This ratio is established based on two key pieces of evidence. First, systematic research conducted by our group in the initial stages, which focused on the preparation and preliminary mechanical property analysis of medium and high-strength HDPE/UHMWPE blend monofilaments, indicates that this ratio achieves an optimal balance between processability and overall mechanical properties [22]. Second, this proportion aligns with the effective addition range, typically reported as 10–30 wt%, that significantly enhances the toughness and load-bearing capacity of polyolefins [23,24]. Consequently, we selected this representative and high-performance ratio for an in-depth investigation of its anti-ultraviolet aging behavior and underlying mechanisms. Table 1 lists the specific sample formulations.

A 1 wt% concentration of polyethylene grafted maleic anhydride (PE-g-MA) was incorporated into the formulation. The polyethylene backbone exhibits compatibility with the matrix, while the maleic anhydride groups facilitate weakly polar interactions. This additive primarily serves as an interface modifier, enhancing interfacial adhesion between the HDPE and UHMWPE phases and promoting the dispersion of UHMWPE within the matrix. Additionally, the inclusion of antioxidants (1010 and 168) aims to mitigate thermal oxidative degradation of the material during processing.

### 2.2. Sample Preparation

High-density polyethylene (HDPE) resin and ultra-high-molecular-weight polyethylene (UHMWPE) powder were dried in a vacuum drying oven at 70 °C for 12 h. Upon completion of the drying process, the raw materials were placed in a high-speed mixer at room temperature according to the proportioning scheme outlined in Table 1. Initially, a low-speed mixing phase of 5 min was performed to facilitate the preliminary combination of the raw materials, followed by 15 min of high-speed mixing to ensure uniform dispersion of all components. The blending and extrusion processes were conducted using a KLD-20 twin-screw extruder (provided by Kailidi New Materials Technology Co., Ltd. Shanghai, China), and the extrudate was subsequently granulated with a pelletizer to produce masterbatches.

The two masterbatches were separately introduced into the ST-CDS30-30 single-screw spinning machine (provided by Shentong Machinery Manufacturing Co., Ltd. Changzhou, Jiangsu, China) for melt spinning. The extrusion temperature was maintained within a range of 170 to 240 °C, with a screw diameter of 30 mm, a length-to-diameter ratio of 30:1, and a screw speed of 50 rpm. The melt was extruded through a spinneret and subsequently cooled and solidified in water at 25 °C to produce primary filaments. These filaments were then drawn in a 98 °C hot water bath using a drawing machine (provided by Shentong Machinery Manufacturing Co., Ltd. Changzhou, Jiangsu, China), resulting in pure HDPE monofilaments and HDPE/UHMWPE blend monofilaments with an approximate diameter of 0.20 mm.

### 2.3. Ultraviolet (UV) Aging Test

Continuous ultraviolet radiation aging experiments were conducted on the samples using an ultraviolet aging test chamber from Zhongya Test Equipment Co., Ltd. Huai’an, Jiangsu, China. The core parameters of this experiment were designed in accordance with the international standard ISO 4892-3:2024 [25], “Plastics—Methods of Exposure to Laboratory Light Sources—Part 3.” Following the relevant guidelines for “Fluorescent Ultraviolet Lamp” and ASTM G154-23 [26], “Standard Practice for Operating Fluorescent Ultraviolet (UV) Lamp Apparatus for Exposure of Materials,” the UVA-340 type fluorescent ultraviolet lamp was selected. Its spectral energy distribution within the wavelength range of 295–365 nm closely aligns with the short-wave band of solar ultraviolet radiation, making it suitable for simulating the photoaging of outdoor materials. The irradiance at a wavelength of 340 nm was maintained at 0.76 W/m^2^, while the temperature and relative humidity within the test chamber were kept constant at 50 °C and 55%, respectively. The experiment employed a continuous illumination mode to investigate the synergistic effects of ultraviolet light and heat. The humidity conditions established in this study align with standard benchmark schemes outlined in relevant accelerated aging protocols, such as ISO 4892-3 and ASTM G154. The objective is to create a stable and controllable experimental environment that facilitates the investigation of the synergistic effects of ultraviolet radiation and thermal stress, rather than attempting to replicate the extreme high humidity characteristic of the marine atmosphere. Employing moderate humidity, as opposed to saturated conditions, mitigates the risk of severe surface condensation during the experimental period. This approach reduces the potential for complex secondary reactions that may arise from the presence of an aqueous phase, thereby allowing for a clearer analysis of the predominant chain of photo-thermal oxidation.

Prior to the commencement of the ultraviolet aging test, pure HDPE and HDPE/UHMWPE monofilaments, which have been spun and drawn, are pre-cut into independent samples with an effective length of 700 mm. For each material, a minimum of five parallel samples is prepared for each designated aging time point (0 h, 150 h, 300 h, 450 h, 600 h). All pre-prepared samples are simultaneously placed in the ultraviolet aging chamber for continuous irradiation. At each specified aging time point, the corresponding group of samples is removed directly from the aging chamber. Mechanical property assessments and other characterization tests are conducted immediately, without any secondary cutting or heat treatment. This procedure ensures that samples at different aging time points maintain identical initial states and preparation histories, thereby allowing any observed performance changes to be strictly attributed to the duration of ultraviolet radiation.

### 2.4. Analysis Methods

Scanning electron microscope (SEM): The surface morphology of the test samples was analyzed using the JSM-6390LA scanning electron microscope from Electronics Co., Ltd. Akishima, Tokyo, Japan. Samples of varying ages were sectioned into approximately 1 cm pieces and adhered to conductive tape. Subsequently, the samples were coated with gold to enhance their conductivity. The working voltage was set to 10 kilovolts, and images of each sample were captured at two magnifications: 50 μm and 10 μm.

Infrared Spectroscopy (FTIR): The Spectrum Two Fourier transform infrared spectrometer from Perkin Elmer, Waltham, MA, USA, was employed to analyze the surface chemical structure of the test sample using the attenuated total reflection mode. The test wavelength range spanned from 500 to 4000 cm^−1^, with a resolution of 4 cm^−1^. The FTIR technology utilized in this study has a detection depth of approximately 2 μm and primarily captures the chemical information at the material’s surface. This characteristic provides a critical window for observing the initiation of oxidation reactions in the photochemical process of ultraviolet aging, which commences at the surface. To quantitatively assess the degree of oxidation, the spectra underwent baseline correction, followed by the calculation of the carbonyl index (*CI*) (1) based on the carbonyl peak area at 1745 cm^−1^ and the unsaturated index (*UI*) (2) derived from the unsaturated peak area at 1640 cm^−1^. Both indices utilized the methylene bending vibration peak at 1472 cm^−1^ as the internal standard.

(1) $$CI=\frac{A_{1745}}{A_{1472}}\times 100\%$$

(2) $$UI=\frac{A_{1640}}{A_{1472}}\times 100\%$$

In the formula, $A_{1745}$ is the peak area at 1745 cm^−1^, $A_{1640}$ is the peak area at 1640 cm^−1^, and $A_{1472}$ is the peak area at 1472 cm^−1^.

Thermogravimetric analysis (TGA): The thermal stability of the samples was evaluated using the TGA4000 thermogravimetric analyzer from Perkin Elmer, Waltham, MA, USA. A sample weighing 5–10 mg was placed in an alumina crucible. Under a nitrogen atmosphere, the sample was heated at a rate of 20 °C/min from 30 °C to 600 °C. Nitrogen was chosen to examine the changes in the intrinsic thermal stability of the material following ultraviolet aging. This inert environment effectively prevents new thermal oxidation reactions during testing, ensuring that the observed thermal decomposition behavior is directly attributable to the internal structural damage induced by ultraviolet aging. By analyzing the TGA data and its differential curve (DTG), the following characteristic temperatures were determined. *T*_5%_ and *T*_10%_ correspond to the temperatures at which the weight loss rates reach 5% and 10%, respectively, and are used to characterize the initial decomposition behavior. *T_max_* denotes the temperature at which the peak maximum weight loss rate occurs on the DTG curve, reflecting the temperature at which the polymer main chain skeleton experiences the most intense thermal decomposition, thereby indicating the thermal stability of its primary structure.

Thermal performance analysis (DSC): The DSC 204F1 differential scanning calorimeter from NETZSCH, Selb, Bavaria, Germany, was utilized to perform thermal performance analysis on the tested samples. Each sample, with a mass ranging from 5 to 10 mg, was placed in an aluminum crucible. The analysis was conducted under a nitrogen flow rate of 50 mL/min, involving a cycle of heating and cooling at a rate of 10 degrees Celsius per minute. The temperature range for the experiment was established from 30 °C to 180 °C. This study utilizes a single heating cycle to directly capture and compare the melting and crystallization behaviors of samples in their ‘current state,’ which encompasses the entire processing and aging history, following ultraviolet aging for varying durations. All samples undergo a uniform preparation and testing protocol. Consequently, the relative changes in melting temperature (*T_m_*) and crystallinity (*X_c_*) effectively reflect the cumulative effects of the aging process on the crystal structure, superimposed on the shared processing history. The crystallinity of the material is calculated according to Formula (3):

(3) $$X_{c}=\frac{\Delta H_{m}}{\Delta H_{m}^{0}}\times 100\%$$

In the equation, $\Delta H_{m}$ represents the measured melting enthalpy, while $\Delta H_{m}^{0}$ denotes the melting enthalpy of fully crystallized HDPE (100%) (293 J/g) [27].

Mechanical tensile test: All monofilament samples underwent mechanical property assessments using the Instron-4466 strength testing machine (Instron, High Wycombe, Buckinghamshire, UK). The testing adhered to the GB/T 14344-2022 standard [28], “Test Method for Tensile Properties of Chemical Fiber Filaments.” Testing conditions included a temperature of 25 °C and a humidity level of 62%. The tensile speed was set at 250 mm/min, and S-shaped fixtures were employed, with the effective sample length between the fixtures measuring 700 mm. The primary mechanical parameters of the sample, including yield strength, breaking strength, and elongation at break, were derived from the recorded stress–strain curves. In this study, yield strength was determined using the 0.2% strain offset method, while breaking strength was identified as the maximum stress value experienced by the specimen during the tensile test, corresponding to the peak value of the stress–strain curve. A minimum of five valid samples were tested for each group, and the coefficient of variation was maintained within 5. The results are presented as mean ± standard deviation. The stretching speed is determined by the quasi-static stretching conditions specified in the standard. This standard rate is selected to create a consistent benchmark for evaluating the performance of all samples under strictly controlled and repeatable laboratory conditions. Such an approach ensures that the measured strength and elongation can be distinctly attributed to the variable of ultraviolet aging, thereby offering a reliable foundation for elucidating the relative superiority or inferiority of the material’s anti-aging performance.

## 3. Results

### 3.1. SEM Analysis

**Figure 1:**
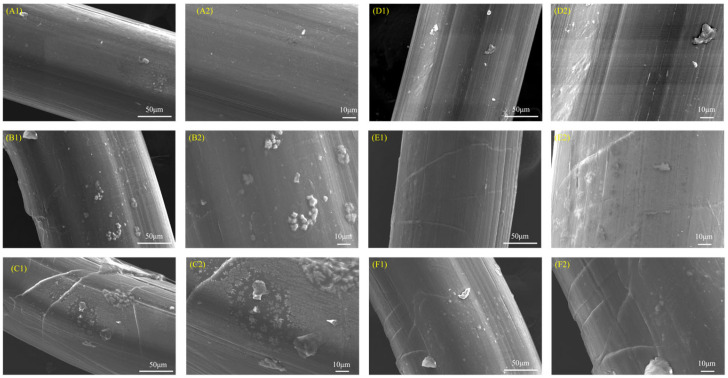
SEM images of surface morphology for HDPE and HDPE/UHMWPE blend monofilaments at different aging times. (**A1**,**A2**) Pure HDPE, 0 h; (**B1**,**B2**) Pure HDPE, 300 h; (**C1**,**C2**) Pure HDPE, 600 h; (**D1**,**D2**) HDPE/UHMWPE blend monofilament, 0 h; (**E1**,**E2**) Blended filament, 300 h; (**F1**,**F2**) Blended filament, 600 h. The image labeled “1” has a scale bar of 50 μm, while the image labeled “2” has a scale bar of 10 μm.

Scanning electron microscopy (SEM) employs signals generated by electron scanning of the sample surface to produce images that visually depict changes in surface morphology of fine materials. Systematic observations of the surface morphology alterations in pure HDPE and HDPE/UHMWPE blend monofilaments after 0 h, 300 h, and 600 h of ultraviolet radiation were conducted using SEM, with results presented in Figure 1. The left column (A1, B1, C1, D1, E1, F1) of the figure illustrates the overall morphology at a scale of 50 μm, while the right column (A2, B2, C2, D2, E2, F2) displays the local fine structure at a scale of 10 μm. Before aging (0 h), both types of monofilaments exhibited smooth and dense surfaces. The surfaces of pure HDPE monofilaments (Figure 1(A1,A2)) and HDPE/UHMWPE blend monofilaments (Figure 1(D1,D2)) displayed only minor fine marks resulting from the processing. After aging for 300 h, the pure HDPE monofilament samples exhibited surface roughness and localized bulging within a 50 μm field of view (Figure 1(B1)). High-magnification images (Figure 1(B2)) revealed distinct microcracks and bulging structures interspersed among them. In contrast, the blended monofilaments, after the same aging period (Figure 1(E1,E2)), maintained a continuous overall macroscopic profile, showing no significant cracks. However, a closer examination at higher magnifications (Figure 1(E2)) indicated that the original processing streaks along the axial direction on the surface were no longer as uniform and smooth as in the initial state. Additionally, discontinuous point protrusions and extremely fine, short transverse microcracks began to emerge. After 600 h of aging, the degradation of pure HDPE intensified markedly (Figure 1(C1,C2)), resulting in numerous wide cracks distributed alternately across the surface. The raised structures coalesced and expanded, with some areas exhibiting signs of spalling. In contrast, the density of point defects in the blended monofilaments (Figure 1(F1,F2)) increased relative to the 300 h mark; the axial fringes in localized regions became blurred, and the number of microcracks increased slightly, although their propagation remained limited. It is important to note that the magnification of the existing SEM images primarily illustrates the initiation and macroscopic propagation of cracks, offering limited insight into the evolution of surface roughness at the nanoscale. Nevertheless, compared to the extensive, interlaced cracking and peeling observed in pure HDPE samples after the same aging duration, the surface morphology evolution of the blended monofilaments was significantly delayed.

### 3.2. FTIR Analysis

**Figure 2:**
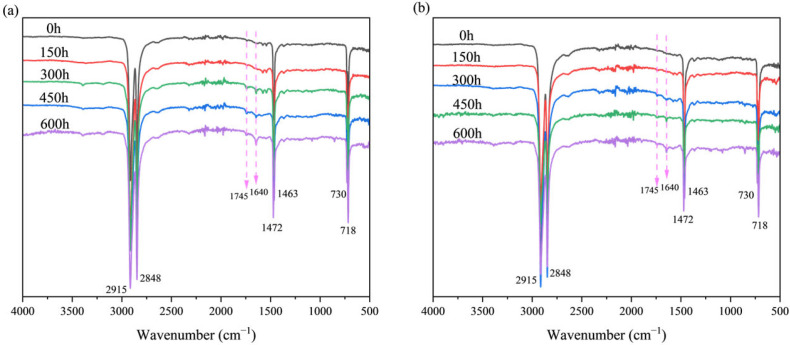
FTIR spectra of HDPE monofilaments (**a**) and HDPE/UHMWPE blend monofilaments (**b**) after different UV aging durations.

**Figure 3:**
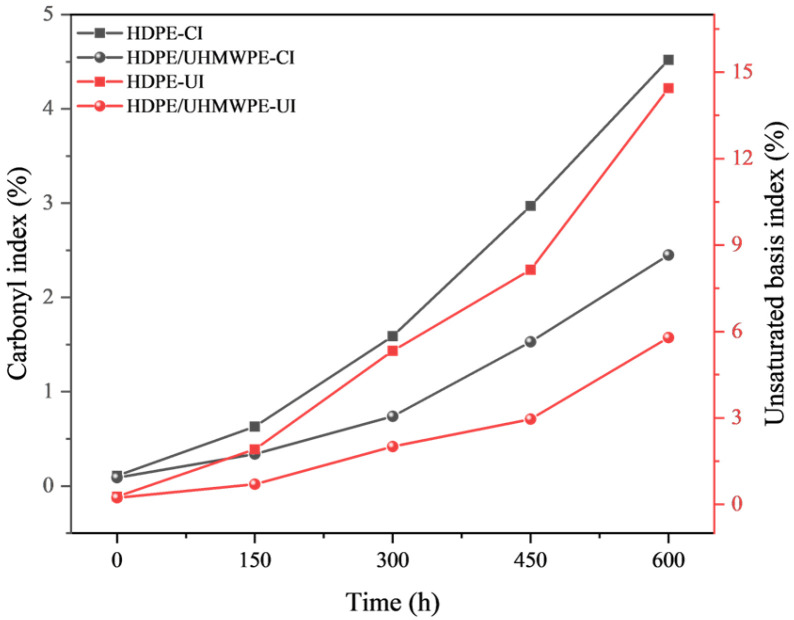
Carbonyl index (*CI*) and unsaturated group index (*UI*) of each sample under different aging times.

To investigate the chemical alterations occurring on the sample surface during the aging process, we systematically analyzed the surface structural changes in pure HDPE monofilaments and HDPE/UHMWPE blend monofilaments subjected to varying durations of ultraviolet aging using Fourier transform infrared spectroscopy (FTIR). The results are presented in Figure 2. Throughout the aging process, all samples exhibited six characteristic absorption peaks that correspond to the primary chain structure of polyethylene. These peaks are located at 2915 cm^−1^ (CH_2_ asymmetric stretching vibration), 2848 cm^−1^ (CH_2_ symmetrical stretching vibration), 1472 cm^−1^ and 1463 cm^−1^ (CH_2_ bending vibration), and 730 cm^−1^ and 718 cm^−1^ (CH_2_ in-plane swing vibration) [29]. The peak positions and intensities of these features did not exhibit significant shifts or attenuation under ultraviolet radiation for up to 600 h, and the C-H skeletons of the main chains of both materials remained largely intact throughout the aging process. As ultraviolet aging progressed, new absorption peaks emerged near 1745 cm^−1^ and 1640 cm^−1^. The absorption peak at 1745 cm^−1^ corresponds to the stretching vibration of the carbonyl group (C=O), which serves as a typical indicator of the oxidation of polyethylene chains into oxygen-containing products such as ketones, aldehydes, and carboxylic acids [30,31]. The absorption peak at 1640 cm^−1^ is associated with the stretching vibration of the C=C double bond, indicative of the unsaturated terminal group generated by molecular chain breakage [32,33]. Comparative analysis of the carbonyl index (*CI*) and unsaturated index (*UI*) for the two samples (Figure 3) reveals that the *CI* and *UI* of pure HDPE monofilament exhibit exponential increases with aging time, particularly accelerating after 300 h. In contrast, the growth rates of *CI* and *UI* for HDPE/UHMWPE blend monofilaments remain consistently lower throughout the aging cycle compared to those of the HDPE monofilaments. After 600 h of aging, the *CI* and *UI* values for pure HDPE are approximately 1.8 and 2.5 times greater than those of the blended monofilaments, respectively.

### 3.3. DSC Analysis

**Figure 4:**
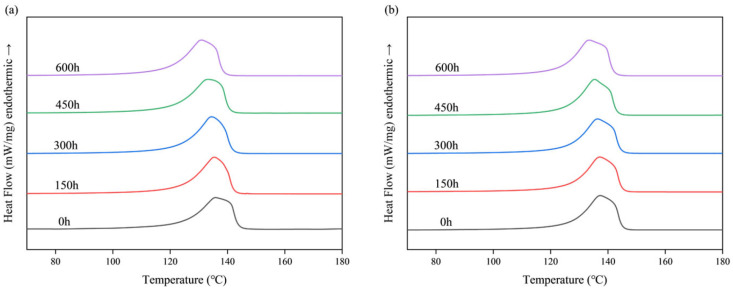
DSC curves of HDPE and HDPE/UHMWPE blend monofilaments at different aging times: (**a**) Pure HDPE monofilament; (**b**) HDPE/UHMWPE blend monofilament.

Differential scanning calorimetry (DSC) was employed to investigate the effects of aging on the thermal and crystallization properties of materials. Both pure HDPE monofilaments and HDPE/UHMWPE blend monofilaments were subjected to DSC analysis following aging, with the results presented in Figure 4 and Table 2. As the duration of ultraviolet aging increased, the melting temperatures (*T_m_*) of both samples exhibited a gradual decline (Table 2). The melting temperature (*T_m_*) of pure HDPE monofilament decreased from an initial value of 135.82 °C to 130.91 °C after 600 h, reflecting a reduction of approximately 4.91 °C. In contrast, the *T_m_* of the HDPE/UHMWPE blend monofilament decreased from 137.33 °C to 133.56 °C, resulting in a reduction of approximately 3.77 °C. Notably, the *T_m_* of the blend samples at each aging time point remains higher than that of pure HDPE. This observation suggests that the blend system maintains a higher melting temperature throughout the aging process, potentially due to differences in its aggregated state structure. In contrast to the trend observed in *T_m_*, both the melting enthalpy (Δ*H_m_*) and the crystallinity (*X_c_*), calculated from it, of the two samples significantly increased with prolonged aging time (Table 2). The crystallinity of pure HDPE rose from an initial 62.35% to 68.26% after 600 h, while that of the blend samples increased from 67.37% to 74.81%. Throughout the aging process, the blend samples consistently exhibited higher *T_m_* and *X_c_* values.

### 3.4. TGA

**Figure 5:**
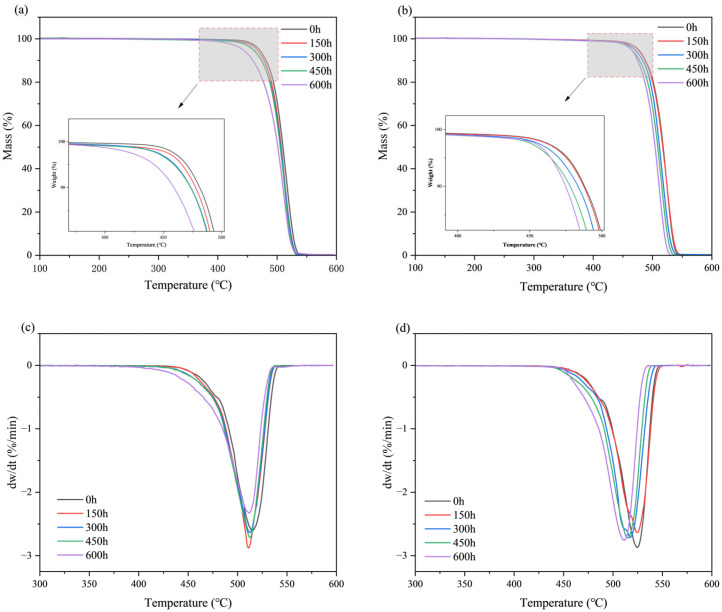
TGA and DTG curves of HDPE and HDPE/UHMWPE blend monofilaments at different aging times: (**a**,**c**) Pure HDPE monofilament; (**b**,**d**) HDPE/UHMWPE blend monofilament.

Thermogravimetric analysis (TGA) was used to evaluate the changes in the thermal stability of the materials by monitoring their thermal decomposition. The thermal degradation behavior of pure HDPE monofilaments and HDPE/UHMWPE blend monofilaments, subjected to varying durations of ultraviolet radiation, was examined using thermogravimetric analysis (TGA). The findings are presented in Figure 5 and Table 3. As ultraviolet aging progressed, the thermal stability of pure HDPE monofilaments exhibited a marked decline (Figure 5a,c). According to the data in Table 3, the initial (0 h) *T*_10%_, *T_max_*, and *T*_5%_ values were 470 °C, 481 °C, and 515 °C, respectively. Following 600 h of aging, these three characteristic temperatures decreased to 442 °C, 459 °C, and 510 °C, representing reductions of 28 °C, 22 °C, and 5 °C, respectively. Notably, the *T*_10%_ value, indicating the onset of material decomposition, and the decline in *T*_5%_ were the most pronounced. In contrast to pure HDPE, the blended monofilaments demonstrated enhanced thermal stability and resistance to aging. Data analysis reveals (Table 3) that the initial thermal stability of the blended monofilament was superior, with *T*_10%_, *T_max_*, and *T*_5%_ values at 477 °C, 488 °C, and 525 °C, respectively, at 0 h. After 600 h of ultraviolet aging, these values decreased to 464 °C, 475 °C, and 512 °C, each reflecting a reduction of 13 °C. Notably, after 600 h of aging, the *T_max_* of the blend (512 °C) remains comparable to the initial value of pure HDPE (515 °C). In contrast, the reductions in *T*_5%_ and *T*_10%_ for the blend (13 °C) are significantly smaller than those observed for pure HDPE (28 °C and 22 °C).

### 3.5. Mechanical Properties Analysis

**Figure 6:**
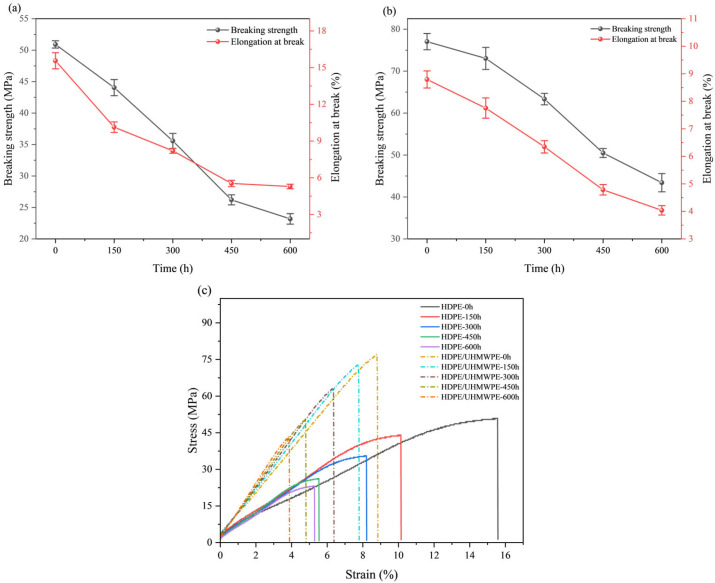
The breaking strength, elongation at break and stress–strain curves (**c**) of HDPE monofilaments (**a**) and HDPE/UHMWPE blend monofilaments (**b**) with different UV aging times.

Mechanical properties represent the ultimate expression of a material’s service capacity. This study investigated the effect of ultraviolet aging time on the mechanical properties of pure HDPE and HDPE/UHMWPE blend monofilaments through mechanical testing. The trends in yield strength, breaking strength, and elongation at break are illustrated in Figure 6 and Table 4. Both materials exhibited a monotonically decreasing trend in yield strength, breaking strength, and elongation at break with increasing ultraviolet aging time; however, the extent of degradation varied significantly. The yield strength of pure HDPE monofilament decreased from an initial value of 45.39 ± 0.41 MPa to 21.79 ± 0.65 MPa after 600 h, reflecting a decay rate of 52.0%. In contrast, the HDPE/UHMWPE blend monofilament demonstrated a higher initial yield strength of 73.86 ± 1.57 MPa, which declined to 43.37 ± 2.06 MPa after 600 h of aging, corresponding to an attenuation rate of 41.3%. The initial breaking strength of pure HDPE monofilament was 50.91 ± 0.58 MPa, which decreased to 23.18 ± 0.83 MPa after 600 h of aging, resulting in an attenuation rate of 54.5%. In contrast, HDPE/UHMWPE blend monofilaments displayed a higher initial strength of 77.03 ± 1.94 MPa, which diminished to 43.41 ± 2.17 MPa after 600 h of aging, yielding a decay rate of 43.7% and indicating a superior strength retention rate. The elongation at break proved to be more sensitive to ultraviolet aging. The elongation of pure HDPE decreased sharply from 15.56 ± 0.66% to 5.29 ± 0.18%, reflecting a loss of 66.0%. Although the initial elongation of the blended monofilament was lower at 8.79 ± 0.31%, it measured 4.04 ± 0.17% after 600 h, resulting in a loss rate of 54.0% and demonstrating a significantly higher retention rate post-aging. The performance degradation curves of both materials display distinct two-stage characteristics. The initial stage, spanning from 0 to 300 h, is marked by rapid degradation, characterized by a relatively high rate of performance loss. In contrast, the subsequent stage, extending from 300 to 600 h, represents a slow attenuation phase, during which the decline in performance begins to stabilize.

## 4. Discussion

This study systematically compares the performance evolution of pure HDPE monofilaments and HDPE/UHMWPE blend monofilaments subjected to simulated ultraviolet accelerated aging. The results indicate a distinct trend: the incorporation of UHMWPE markedly prolongs the overall degradation process of the material, affecting both microstructural and macroscopic properties. The subsequent sections will elucidate these phenomena, investigate their underlying mechanisms, and clarify the significance of this research within both academic and practical contexts. Additionally, this study will objectively identify its limitations and propose future research directions.

### 4.1. From Multi-Scale Performance Degradation to Failure Mode Transformation

This study systematically observed the multi-scale performance of pure HDPE monofilaments and HDPE/UHMWPE blend monofilaments during ultraviolet aging. The findings revealed significant differences in the performance evolution trajectories of the two materials, which ultimately manifested as distinct failure modes.

The performance degradation of pure HDPE monofilaments exhibits accelerated and interconnected characteristics, indicative of rapid brittle failure. In terms of surface morphology, significant microcracks began to emerge after 300 h of aging, and by 600 h, these cracks evolved into extensive, interlaced fissures accompanied by localized spalling (Figure 1(B1,B2,C1,C2)), signifying a swift loss of surface integrity. Fourier Transform infrared spectroscopy (FTIR) analysis of the chemical structure further elucidated its intrinsic deterioration. The primary indicators of oxidation, namely the carbonyl index (*CI*) and the unsaturated index (*UI*), entered a phase of rapid increase following 300 h of aging. The *CI* value surged from 0.63% at 150 h to 4.52% at 600 h (Figure 3), suggesting that the oxidation reaction exhibited a self-accelerating trend in the later stages [34]. The results of the thermal analysis offer a structural explanation for this phenomenon. Differential scanning calorimetry (DSC) indicates that the melting temperature (*T_m_*) has consistently decreased from an initial value of 135.82 °C to 130.91 °C after 600 h. This decline is typically associated with an increase in defects within the crystal structure or a reduction in its overall perfection. Concurrently, the crystallinity (*X_c_*) increased from 62.35% to 68.26% (Table 2), which may be attributed to the further reorganization and recrystallization of the molecular chain segments during the heating test, following chain breakage in the amorphous region [30]. Thermogravimetric analysis (TGA) corroborated a simultaneous deterioration in overall thermal stability. The *T*_5%_ of pure HDPE monofilament decreased significantly by 28 °C (Table 3), providing clear evidence of extensive oxidation on its surface and the formation of numerous weak bonds. The reduction in *T_max_* (Table 3) signifies that aging-induced chain breaks have adversely impacted the overall average molecular weight of the material and the integrity of the main chain skeleton [35]. The attenuation of macroscopic mechanical properties is both intuitive and pronounced. The yield strength, breaking strength, and elongation at break decrease by 52.0%, 54.5%, and 66.0%, respectively, over a period of 600 h (Figure 6, Table 4). These interrelated and progressively amplified chain deteriorations, spanning surface morphology, chemical structure, aggregated state thermal stability, and macroscopic mechanical properties, collectively illustrate the rapid and irreversible brittle failure pathway of pure HDPE. To elucidate the correlation among the aforementioned multi-scale performance degradations, it is essential to clarify the spatial information hierarchy as reflected by various characterization techniques. The FTIR analysis conducted in this study primarily captured the chemical oxidation information of the material’s outermost layer (approximately 2 μm), which serves as the initiation point for microscopic damage induced by photoaging. In contrast, DSC and TGA are more sensitive to the bulk aggregation state structure and overall thermal stability of materials. For pure HDPE, the rapid accumulation of surface chemical oxidation and the accelerated loss of bulk structural integrity exhibit a high degree of synchronization, confirming that its aging and deterioration manifest as a “rapid brittle failure” mode that connects the surface to the interior across all scales.

In stark contrast, HDPE/UHMWPE blend monofilaments demonstrate significant performance degradation buffering effects across all observation scales. Their surface morphology remains largely intact. Following aging, only short and sparse microcracks are observed, with no evidence of large-scale cracking or spalling (Figure 1(E1,E2,F1,F2)). FTIR spectral analysis reveals that the chemical oxidation process is markedly delayed. The growth of the carbonyl index (*CI*) is consistently slow, increasing from 0.34% at 150 h to 2.45% at 600 h (Figure 3). The carbonyl index growth in the blend monofilament is substantially lower than that of pure HDPE, providing quantitative evidence that the incorporation of UHMWPE effectively hinders oxygen diffusion and free radical chain reactions. Consequently, the oxidation kinetics transition from a “self-accelerating” mode to a “slow linear” mode [21]. The differential scanning calorimetry (DSC) results further corroborate this observation at the structural level. The melting temperature (*T_m_*) of the blended monofilament decreased from 137.33 °C to 133.56 °C, reflecting a modest reduction. Additionally, at each time point, the *T_m_* remained higher than that of pure HDPE during the same period, suggesting improved thermal stability as indicated by its melting behavior. Although the crystallinity (*X_c_*) increased from 67.37% to 74.81%, this change occurred gradually, building upon its already elevated initial crystallinity (Table 2). Thermogravimetric analysis (TGA) also indicates enhanced overall thermal stability, as evidenced by a 5% decrease in T5 (at 13 °C), which is significantly smaller than that observed for pure HDPE. The coordinated “buffering” achieved across multiple scales—encompassing morphology, chemistry, structure, and thermal stability—contributes to the remarkably gentle attenuation curve of its macroscopic mechanical properties. After 600 h of aging, the material retains a breaking strength of 56.4%, demonstrating a performance retention rate significantly higher than that of pure HDPE.

The disparity in aging behavior between the two materials signifies a fundamental change in the failure mode. Pure HDPE monofilaments exhibit a “rapid brittle failure” pathway that initiates with accelerated chemical oxidation. This process leads to the destruction of the structure and defective reorganization, which subsequently causes rapid expansion of surface cracks. Ultimately, this results in a “cliff” decline in mechanical properties, with each stage exacerbating the others. In contrast, the incorporation of UHMWPE systematically mitigates the entire attenuation chain, from chemical initiation and structural evolution to morphological damage and mechanical loss. This transformation alters the aging response of the material to a “progressive slow deterioration” mode, characterized by coordinated performance across all scales and gradual attenuation. The clear pattern transformation observed at the phenomenological level, supported by multi-dimensional data, serves as the foundation for a more in-depth investigation into the underlying physical mechanisms.

### 4.2. The Dual Physical Mechanisms and Academic Value of UHMWPE Enhancement

#### 4.2.1. The Synergistic Enhancement Mechanism of Physical Hindrance and Structural Skeleton

The systematic characterization results of this study indicate that the exceptional anti-ultraviolet aging performance of HDPE/UHMWPE blend monofilaments arises from the incorporation of the UHMWPE phase. This phase, characterized by its unique ultra-long molecular chain structure, establishes a dual synergistic enhancement mechanism involving “physical hindrancy” and “structural skeleton” within the HDPE matrix [35]. Consequently, the aging response pathway and failure mode of the material have been altered, facilitating a transition from “rapid brittle failure” to “progressive slow deterioration.”

The physical hindrance effect manifests primarily in two ways: delaying oxidation and suppressing crack propagation. First, the extremely high molecular weight of UHMWPE allows for the formation of highly entangled, dense micro-regions. These micro-regions serve as physical barriers that effectively impede the diffusion of oxygen and free radicals [36], thereby inhibiting the photo-oxidative aging chain reaction at its source. The substantial reduction in the growth of the carbonyl index (*CI*) and unsaturated index (*UI*) in the FTIR spectrum (Figure 3) provides direct confirmation of this effect. Second, these rigid micro-regions can pin, deflect, and bridge the micro-cracks that originate in the HDPE phase, thereby consuming fracture energy and preventing their propagation and expansion [37]. The SEM images illustrate that the surface cracks of the co-mixed monofilaments are finer, shorter, and discontinuous (Figure 1(E1,E2,F1,F2)), offering intuitive evidence for this phenomenon. The structural skeleton function provides continuous mechanical support to the material during the later stages of aging. The DSC and TGA results demonstrated that the blend monofilaments exhibited a higher melting temperature (*T_m_*) and a slight decrease in the initial thermal decomposition temperature (*T*_5%_) throughout the aging process (Table 2 and Table 3), alongside a significantly improved retention rate of macroscopic mechanical properties (Figure 6). These thermal performance data indirectly suggest that the UHMWPE phase may have established a more stable physical network, which effectively preserves the overall structural integrity of the material. Moreover, Zaharescu et al. [38] demonstrated that γ irradiation enhances the stability of the crystalline structure in LDPE/UHMWPE blends. A similar trend was observed in the ultraviolet light oxidation system, which more closely resembles natural aging. It is proposed that the dispersed stable crystal regions of UHMWPE may serve as heterogeneous nucleation sites during aging, facilitating the ordered recrystallization of broken HDPE segments and thereby aiding in the preservation of the material’s overall structural integrity. More importantly, when ultraviolet aging leads to the degradation of the molecular chains within the HDPE matrix, resulting in a decline in its load-bearing capacity, dispersed and stable UHMWPE micro-regions establish a persistent secondary stress-bearing network [39]. This network continues to effectively transfer and distribute the load, which accounts for the ability of the blended monofilaments to retain high residual strength and ductility during the later stages of aging (Figure 6). The “physical obstruction” effect postpones the onset of aging, while the “structural skeleton” function mitigates the effects of aging. Together, these mechanisms transform the failure mode of the material from the “rapid brittle failure” characteristic of pure HDPE to “progressive slow deterioration.”

#### 4.2.2. The Microstructure Basis for Realizing the Enhancement Mechanism

The effectiveness of the “physical hindrancy–structural skeleton” collaborative enhancement mechanism is contingent upon a critical microstructural foundation, specifically the complete and uniform dispersion of the UHMWPE phase within the HDPE matrix, along with the stable interface that this dispersion engenders. Uniform dispersion serves as the physical basis for constructing effective reinforcement [21]. In this study, the dispersion of UHMWPE was significantly enhanced by optimizing the melt blending process and incorporating polyethylene grafted maleic anhydride (PE-g-MA) as an interface modifier [40]. It is important to note that in the non-polar HDPE/UHMWPE blend system, PE-g-MA does not achieve “volume increase” through classical chemical reactions. Its polyethylene backbone exhibits favorable thermodynamic compatibility with the two-phase matrix and can become extensively entangled. The weak polarity introduced by the maleic anhydride group enhances interfacial adhesion and wettability between the two phases [41], thereby mitigating the agglomeration of UHMWPE particles and facilitating their uniform dispersion. The regulation of this microscopic morphology is essential for ensuring that the UHMWPE phase can establish a continuous physical barrier. During the prolonged ultraviolet aging process, the interface of this blend system exhibited remarkable functional stability. The sustained benefits of macroscopic mechanical properties (Figure 6) and the significant reduction in surface crack propagation (Figure 1) collectively indicate that stress transmission and crack pinning effects are effectively preserved throughout the aging cycle. Furthermore, the two-phase interface does not serve as a weak link susceptible to failure. This observation suggests that the interface formed through PE-g-MA modification and process optimization is robust enough to withstand the chemical and physical stresses induced during aging, thereby ensuring the long-term efficacy of the UHMWPE reinforcing phase. Consequently, a two-phase system that is uniformly dispersed and stably integrated through process optimization and interface modification fundamentally guarantees the sustained reinforcing effect of the dual synergistic mechanism of “physical hindering–structural skeleton” of UHMWPE [41]. Furthermore, it is essential to examine the role of the composite antioxidant within the system. Throughout the prolonged ultraviolet aging process, the chemical protective effects of antioxidants and the physical reinforcing properties of UHMWPE are inextricably linked. A plausible hypothesis is that the dense entanglement network formed by UHMWPE may physically impede the migration of antioxidant molecules, thereby prolonging their availability and potentially creating a synergistic protective effect in conjunction with the physical network. Consequently, a comprehensive explanation of the enhanced aging performance should be framed within a context where the efficacy of physical barriers and chemical stabilizers is potentially optimized in a synergistic manner.

#### 4.2.3. The Advancement of Existing Research Approaches and Theoretical Deepening

To elucidate the academic contribution of this work, it is essential to analyze the aforementioned mechanism within the context of existing research. The primary observational findings of this study align with previous investigations [42], offering a systematic mechanistic explanation and experimental validation for the empirical understanding of UHMWPE’s role in enhancing the durability of polyolefins. For example, research demonstrates that UHMWPE fibers also exhibit patterns of molecular chain breakage and strength reduction due to ultraviolet radiation in marine environments [43], which corresponds with the aging onset identified in this study. Nevertheless, regarding the depth of material design concepts and mechanistic explanations aimed at performance enhancement, this study diverges significantly from mainstream research approaches, thereby underscoring its unique contribution. In contrast to conventional high-content blends or specialized processing strategies designed to address processing challenges and enhance performance [14,44], this study illustrates that utilizing standard melt blends with moderate to low addition levels of 20 wt% and optimizing dispersion through interfacial compatibilizers can effectively regulate the failure modes of materials solely via physical mechanisms. This method prioritizes the equilibrium among processing universality, cost-effectiveness, and long-term durability, thereby offering a novel perspective for material design aimed at large-scale engineering applications. In contrast to the additive approach commonly employed in industry, which relies on small-molecule chemical reactions (such as HALS) [45], the UHMWPE physical blending strategy presented in this study offers a fundamentally different protective paradigm. Additives function by capturing free radicals or absorbing ultraviolet photons; however, they possess inherent limitations, as their effectiveness diminishes due to migration and consumption. This study establishes a permanent physical entanglement network, creating a durable barrier derived from the material’s structure itself, thereby fundamentally mitigating the environmental release risks associated with small-molecule additives. This approach does not merely replace traditional methods; rather, it provides a complementary and more sustainable solution for applications that impose stringent requirements for long-term environmental safety and service life predictability. The most significant contribution of this research lies in its multi-scale characterization and mechanism analysis, which elucidate for the first time that low-content UHMWPE can actively reshape the aging failure pathway of the HDPE matrix through the synergistic mechanism of “physical obstruction–structural skeleton.” This advancement shifts the understanding of the system from a macroscopic performance comparison of “whether it is effective” to a mechanistic correlation level of “why and how it is effective,” thereby offering a novel theoretical perspective and experimental foundation for designing long-lasting fishery materials with predetermined failure behavior by precisely regulating the morphological structure of heterogeneous polymers.

### 4.3. Application Value and Comprehensive Benefit Assessment

Mechanical properties directly reflect the long-term service capacity of fishery materials. Blended monofilaments demonstrate a mechanical property retention capacity that significantly surpasses that of pure HDPE. After 600 h of ultraviolet aging, the yield strength, breaking strength, and elongation at break of pure HDPE monofilaments decreased significantly by 52.0%, 54.5%, and 66.0%, respectively, exhibiting characteristics of “rapid brittle failure.” In contrast, the performance degradation of blended monofilaments was notably less severe, which can be attributed to the synergistic enhancement mechanism of “physical hindrance–structural skeleton” established within this system. From the perspective of practical engineering applications in fishery materials, the performance characteristics of this blended system, which include “high initial yield and breaking strength, excellent strength retention, and moderate initial elongation,” demonstrate a practical and optimized engineering balance. A higher yield strength retention rate indicates that the material can effectively withstand the relaxation and deformation of fishing gear nodes after prolonged use, which is crucial for preserving the geometric integrity and structural stability of the fishing gear. Although its initial elongation at break is lower than that of pure HDPE, the initial breaking strength of the blended material is approximately 51% higher, thus providing a greater initial safety load reserve for the net equipment. After 600 h of aging, the elongation rates of both materials converge; however, the residual strength of the blended monofilament remains significantly higher than that of pure HDPE. This finding indicates that the blend possesses a superior load-bearing safety margin during the later stages of long-term service. Consequently, while the blended material sacrifices some initial flexibility, it achieves a more predictable and gradual deterioration pattern, along with enhanced overall mechanical reliability throughout its entire life cycle. This performance balance strategy is not only acceptable but also offers considerable advantages in fishery applications.

To translate the aforementioned performance advantages into quantifiable durability indicators, we conducted a preliminary assessment of the material’s potential to extend service life. Based on the rate of breaking strength decay under identical aging conditions dominated by ultraviolet rays, we estimate that its theoretical service life could exceed that of pure HDPE by more than 25%. This calculation elucidates the relative advantage of the blend system in resisting ultraviolet aging and provides a quantitative reference for its application in environments characterized by photoaging. It is important to note that this potential for life extension is derived from theoretical relative values obtained through single-factor accelerated tests conducted in the laboratory. The actual marine environment, however, is a complex system influenced by multiple stressors. The service life of materials is not solely determined by ultraviolet radiation; it is also intricately linked to various factors, including temperature, humidity, salinity, and mechanical load. Furthermore, these factors may interact synergistically, potentially accelerating the aging process. To convert laboratory data into dependable life predictions for real-world applications, it is essential to integrate the failure criteria under specific conditions and validate them through multi-factor coupling accelerated aging experiments or extended real-sea exposure tests. Nevertheless, the comprehensive understanding of physical enhancement mechanisms and failure mode transitions presented in this study offers vital insights for future material design and the development of life assessment models in actual marine environments.

This HDPE/UHMWPE blend material, noted for its exceptional performance retention and significant potential for life extension, offers three environmental benefits throughout its lifecycle. First, it facilitates a reduction in plastic waste at the source. By extending the material’s lifespan by over 25%, the frequency of fishing gear replacement is directly decreased. For instance, in the case of a typical medium-sized aquaculture cage utilizing approximately 500 kg of polyethylene, employing this blend can result in a reduction of about 100 kg of plastic waste over the same service life, thereby mitigating the impact of marine plastic pollution at its origin. Second, it enables process control over microplastic release. The blend’s superior resistance to crack propagation significantly delays the onset of microscopic surface damage and macroscopic cracking. This characteristic effectively mitigates the generation of secondary microplastics resulting from brittle fracture, surface powdering, and friction wear at the material design stage, thereby diminishing the risk of their ongoing release into marine environments. Ultimately, it embodies comprehensive sustainability throughout the entire life cycle. Although the initial cost of raw materials has risen due to the incorporation of UHMWPE, the extended service life has decreased the material usage cost over time. The reduced burden of waste disposal, potential expenses associated with ecological restoration, and economic losses to fisheries resulting from premature damage to fishing gear collectively represent a significant comprehensive benefit, aligning with the core principles of a circular economy and sustainable development.

### 4.4. Limitations and Future Prospects

#### 4.4.1. The Gap Between Single-Factor Aging in the Laboratory and the Real Marine Environment

The ultraviolet accelerated aging experiment conducted in this study adhered to ISO and ASTM standards, with its spectrum aligned to the short-wave band of sunlight’s ultraviolet light. This approach seeks to efficiently and reproducibly elucidate the performance evolution and the relative advantages and disadvantages of materials subjected to ultraviolet light, the primary aging factor, under controlled conditions. Consequently, it provides a solid foundation for interpreting mechanisms and screening materials. The single-factor experimental model employed in this study represents a necessary simplification of the intricate real marine environment. Notably, the constant moderate humidity (55%) used in the experiment markedly differs from the long-term, near-saturated high humidity characteristic of the actual marine atmosphere. The real marine environment functions as a dynamic system shaped by the prolonged interaction of multiple factors, including ultraviolet radiation, salt spray erosion, damp heat cycles, and wave loads. These factors can produce synergistic effects, such as the catalytic influence of salt on oxidation reactions and the physical stress induced by alternating dry and wet conditions. However, the current experimental conditions do not replicate these critical processes. Consequently, there exists inherent uncertainty in extrapolating conclusions derived from a single ultraviolet aging experiment to complex marine environments. The precise quantitative relationship between laboratory acceleration conditions and real-world environments must be calibrated using subsequent outdoor exposure data. Consequently, the conclusions drawn in this study regarding the advantages and mechanisms of the anti-ultraviolet aging performance of the blend system are valid only under a singular ultraviolet stress condition in the laboratory. However, when attempting to extrapolate and accurately predict the absolute service life of the material within the complex and multifactorial marine environment, inherent uncertainties arise. To bridge the divide between laboratory research and engineering applications, future efforts should systematically conduct multifactor (ultraviolet/salt spray/damp heat/load) coupled accelerated aging experiments. Ultimately, these experiments should verify and calibrate the laboratory model through long-term real-sea exposure tests, thereby establishing a more reliable method for predicting service life.

#### 4.4.2. Decoupling of the Interaction Between Formula Components and Thermal Effects

In this study, a uniform dose of the compound antioxidant was incorporated into both pure HDPE and its blends to standardize the initial thermal oxidation state across all samples. However, a significant limitation of this research is that the current experimental design fails to accurately isolate the independent contributions of the chemical protective effects of antioxidants from the physical enhancement provided by UHMWPE during prolonged ultraviolet aging. Furthermore, there is a notable absence of direct experimental evidence regarding the consumption of antioxidants in blends, such as tracking the characteristic peaks of antioxidants via FTIR or analyzing their concentration decline through HPLC. Additionally, it is plausible that the dense entanglement network formed by UHMWPE may impede the migration and consumption of antioxidant molecules, thereby indirectly prolonging their protective effect. Consequently, while multi-scale data, including SEM, DSC, and TGA, strongly suggest that the physical structure of UHMWPE is crucial in delaying aging, the existing data do not entirely exclude the possibility that antioxidants play a significant or synergistic role in this process. The 50 °C aging temperature utilized in the experiment accelerates the aging process; however, it also introduces thermal stress and ultraviolet radiation, complicating the quantification of the effects of pure thermal oxygen aging. A fundamental strategy involves conducting full-factor experiments to systematically prepare pure HDPE and blended samples, both with and without antioxidants. Parallel experiments will be performed under two distinct conditions: a 50 °C anechoic chamber for pure thermal oxygen aging and 50 °C ultraviolet irradiation for photo-thermal coupling aging. This experimental framework will enable direct quantification of the intrinsic anti-aging contributions of UHMWPE, elucidate the consumption kinetics of antioxidants across different matrices, and distinctly isolate the independent effects of thermal stress on the aging process. Consequently, this approach will provide a robust foundation for optimizing material stability in complex marine environments.

#### 4.4.3. Deepening of Microscopic Mechanism, Empirical Research and Service Performance Evaluation

This study employs characterization methods including SEM, FTIR, DSC, and TGA to systematically elucidate the multi-scale evolution of materials, encompassing surface morphology, chemical structure, thermal properties, and macroscopic mechanical properties during ultraviolet aging. Consequently, it proposes an explanatory model grounded in the physical strengthening mechanism of UHMWPE. While this model effectively accounts for the evolution of macroscopic performance, it necessitates more direct experimental evidence to quantitatively describe the key structural parameters involved, such as entanglement density, interface strength, and microscopic load transfer paths. To advance the explanation of the mechanism toward quantification and visualization, future research should incorporate characterization techniques with enhanced spatial resolution and increased sensitivity to microstructure. For example, the nanomechanical mapping mode of atomic force microscopy can facilitate direct observation of phase distribution, the evolution of interfacial mechanical properties, and the behavior of microcracks within the blend system. Additionally, wide-angle/small-angle X-ray scattering can accurately analyze crystal structure parameters and the structural evolution of the amorphous region. Dynamic thermomechanical analysis or rheological methods can sensitively characterize the relaxation behavior of molecular chains and the interactions at the phase interface, thereby providing critical evidence to bolster the stability of the proposed mechanism.

There exists a defined interpretive boundary concerning representational means. For example, the distribution of oxidation products, including carbonyl groups and unsaturated bonds, as revealed by ATR-FTIR, is primarily confined to the outermost layer of the material, approximately 2 μm in depth. While this analysis effectively captures the initial region of the aging reaction, a comprehensive evaluation of whether oxidation has penetrated into the bulk phase of the material or an accurate quantification of the average oxidation degree in that phase necessitates future research. Such studies should integrate additional techniques that demonstrate greater sensitivity to the bulk phase, including micro-FTIR surface scanning or specific chemical titration methods. In the DSC analysis, only the first heating cycle was utilized. The melting behavior indicated by this curve reflects the combined effects of the material’s initial processing history and its ultraviolet aging history. By comparing the relative changes in the first heating curves of all samples, which share the same processing history, one can reliably assess the overall trend of the aging process’s impact on the final aggregated state structure of the material. However, this approach presents challenges in precisely distinguishing the “intrinsic crystallization ability changes” induced by aging—such as the effects of molecular chain breakage on recrystallization kinetics—from the “immediate damage to crystal integrity” resulting from aging. To elucidate the influence of ultraviolet aging on the intrinsic mechanisms governing material crystallization behavior, future research should incorporate secondary heating cycle experiments. This would help eliminate the effects of processing history and allow for a more accurate characterization of the changes in molecular chain rearrangement and crystallization ability induced by aging.

In the evaluation of service performance, while quasi-static tensile properties effectively indicate the aging and damage of materials, they fall short in providing a comprehensive assessment of the long-term reliability of fishery materials subjected to actual dynamic loads, such as wave impacts and cyclic traction. It is important to highlight that polyethylene, being a viscoelastic material, exhibits significant strain rate dependence in its mechanical response. The standard tensile rate of 250 mm/min employed in this study, although suitable for comparative research on material screening and aging mechanisms, differs by several orders of magnitude from the high-strain-rate conditions that fishing nets may encounter during actual service, particularly due to dynamic or impact loads like wave impacts and instantaneous hooks. Consequently, limitations exist in directly correlating the current tensile data with the absolute performance of the material under real-world complex working conditions. Impact strength and fatigue performance serve as critical engineering indicators that influence the durability of fishing nets, ropes, and other products in marine environments. Thus, developing a comprehensive application performance evaluation system is an essential area for further research. Future investigations should systematically perform notch impact strength tests and tension-tensile fatigue tests on samples subjected to ultraviolet aging. Additionally, researchers should generate stress-life curves and consider conducting tensile tests at varying strain rates to establish a more robust material performance evaluation system and life prediction model tailored to the actual marine dynamic service environment.

## 5. Conclusions

In this study, fishery monofilaments composed of HDPE/UHMWPE (80/20 wt%) were prepared through melt spinning, and their performance evolution under simulated ultraviolet accelerated aging was systematically assessed. The findings indicate that the incorporation of UHMWPE markedly improves the overall anti-aging properties of the material. After 600 h of aging, only minor and short microcracks were observed on the surface of the blended monofilament, and the increase in the carbonyl index was minimal. The reduction in the initial temperature of thermal decomposition (*T*_5%_) was 13 °C, which is significantly less than the 28 °C observed for pure HDPE. Furthermore, the retention rates of strength and elongation in the blend were considerably higher than those of pure HDPE. The notable enhancement in comprehensive performance is intricately linked to the establishment of a unique “physical hindrance–structural skeleton” mechanism in UHMWPE. Physical hindrances are primarily manifested in the delay of oxidation and the suppression of crack formation. Meanwhile, the structural skeleton effect guarantees continuous mechanical support for the material during the later stages of aging. Additionally, this mechanism may exhibit a synergistic protective effect in conjunction with the antioxidants present in the system, collectively transforming the failure mode of the material from “rapid brittle failure” to “progressive slow deterioration.” This study illustrates that through conventional processing and the judicious incorporation of UHMWPE, a significant enhancement in the material’s anti-aging performance can be achieved. This finding offers a novel physical modification strategy and a theoretical foundation for the development of durable and environmentally friendly polyolefin materials for use in fisheries. Future research should focus on real marine environments, conduct multi-factor coupling aging studies, and further elucidate the mechanisms and application evaluations through microscopic characterization and dynamic mechanical testing.

## Figures and Tables

**Table 1:** Specific sample formulations.

Sample	HDPE (wt%)	UHMWPE (wt%)	PE-g-MA (wt%)	Antioxidant (wt%)
HDPE	100	0	1	0.5
HDPE/UHMWPE	80	20	1	0.5

**Table 2:** DSC Data for HDPE Monofilaments and HDPE/UHMWPE Blends with Different Aging Durations.

Sample	Time (h)	*T_m_* (°C)	△*H_m_* (J/g)	*X_c_* (%)
HDPE	0	135.82	182.7	62.35
	150	135.33	187.3	63.92
	300	134.45	191.9	65.49
	450	133.17	193.3	65.97
	600	130.91	200.0	68.26
HDPE/UHMWPE	0	137.33	197.4	67.37
	150	137.14	200.1	68.29
	300	136.42	205.3	70.07
	450	135.35	211.6	72.22
	600	133.56	219.2	74.81

**Table 3:** Degradation Temperatures of HDPE Monofilaments and HDPE/UHMWPE Blends with Different Aging Durations.

Sample	Time (h)	*T*_5%_ (°C)	*T*_10%_ (°C)	*T_max_* (°C)
HDPE	0	470	481	515
	150	466	478	513
	300	460	474	512
	450	459	473	512
	600	442	459	510
HDPE/UHMWPE	0	477	488	525
	150	475	487	524
	300	470	483	518
	450	465	478	515
	600	464	475	512

**Table 4:** Mechanical property data of HDPE monofilaments and HDPE/UHMWPE blend monofilaments with different ultraviolet aging times.

Sample	Time (h)	Yield Strength (MPa)	Breaking Strength (MPa)	Elongation at Break (%)
HDPE	0	45.39 ± 0.41	50.91 ± 0.58	15.56 ± 0.66
	150	40.13 ± 0.75	44.05 ± 1.28	10.13 ± 0.43
	300	32.36 ± 1.02	35.58 ± 1.20	8.20 ± 0.20
	450	24.48 ± 0.73	26.21 ± 0.80	5.53 ± 0.26
	600	21.79 ± 0.65	23.18 ± 0.83	5.29 ± 0.18
HDPE/UHMWPE	0	73.86 ± 1.57	77.03 ± 1.94	8.79 ± 0.31
	150	69.91 ± 2.41	73.03 ± 2.62	7.75 ± 0.37
	300	62.17 ± 1.30	63.34 ± 1.36	6.35 ± 0.22
	450	50.18 ± 0.95	50.49 ± 1.07	4.79 ± 0.19
	600	43.37 ± 2.06	43.41 ± 2.17	4.04 ± 0.17

## Data Availability

The original contributions presented in this study are included in the article. Further inquiries can be directed to the corresponding author.

## References

[1] Zhang F., Shi J., Zhang J., Zhang W., Li Y., Zhao L., Cao Y., Lu C., Wang S. (2024). Research Status and Prospect of Functional Fishery Materials. J. Fish. China.

[2] Liu C. (2022). Progress of the World’s Plastics Industry in 2020~2021 (I): General Purposed Plastics. China Plast. Ind..

[3] Xu Y., Li J., Wang J., Luo Z. (2022). In-situ Compatibilization of Isotactic Polypropylene and High Density Polyethylene Blends by Co-branching Reaction. China Plast. Ind..

[4] Wei S. (2022). Bending Stiffness Analysis of Plastic Frame of Nearshore Aquaculture Cages. Fish. Mod..

[5] Liu Y., Huang X., Yang K., Xie S., Qi J. (2025). Study on Permeability of Hydrogen in High-density Polyethylene Materials. Plast. Sci. Technol..

[6] Liu S., Zhang R., Fu C., Zheng T., Xue P. (2025). Changes in Heat Resistance and Mechanical Properties of Peroxide Cross-Linking HDPE: Effects of Compounding Cross-Linkers. Polymers.

[7] Jiang X., Gallager S., Pàmies R.P., Ruff S.E., Liu Z. (2024). Laboratory-Simulated Photoirradiation Reveals Strong Resistance of Primary Macroplastics to Weathering. Env. Sci. Technol..

[8] Duan J., Li Y., Gao J., Cao R., Shang E., Zhang W. (2022). ROS-Mediated Photoaging Pathways of Nano- and Micro-Plastic Particles under UV Irradiation. Water Res..

[9] Raota C.S., Lotfi S., Lyubimenko R., Richards B.S., Schäfer A.I. (2023). Accelerated Ageing Method for the Determination of Photostability of Polymer-Based Photocatalytic Membranes. J. Membr. Sci..

[10] Jiang M., Sun X., Sun M., Li Y., Zhu H. (2024). Effect of High Intensity Ultraviolet Irradiation on Properties of Modified Polyethylene. Plast. Sci. Technol..

[11] Fairbrother A., Hsueh H.-C., Kim J.H., Jacobs D., Perry L., Goodwin D., White C., Watson S., Sung L.-P. (2019). Temperature and Light Intensity Effects on Photodegradation of High-Density Polyethylene. Polym. Degrad. Stab..

[12] Cai L., Wang J., Peng J., Wu Z., Tan X. (2018). Observation of the Degradation of Three Types of Plastic Pellets Exposed to UV Irradiation in Three Different Environments. Sci. Total Environ..

[13] Jiang T., Zhang J. (2021). Comparison of UV Resistance of HDPE Added with Hindered Amine Light Stabilizers with Different Molecular Structures. Polym. Adv. Techs.

[14] Patel K., Chikkali S.H., Sivaram S. (2020). Ultrahigh Molecular Weight Polyethylene: Catalysis, Structure, Properties, Processing and Applications. Prog. Polym. Sci..

[15] Wang H., Xu L., Li R., Hu J., Wang M., Wu G. (2016). Improving the Creep Resistance and Tensile Property of UHMWPE Sheet by Radiation Cross-Linking and Annealing. Radiat. Phys. Chem..

[16] Hu P., Cheng Y., Zhang P., Liu J., Yang H., Chen J. (2021). A Metal/UHMWPE/SiC Multi-Layered Composite Armor against Ballistic Impact of Flat-Nosed Projectile. Ceram. Int..

[17] Pang W., Ni Z., Wu J., Zhao Y. (2018). Investigation of Tribological Properties of Graphene Oxide Reinforced Ultrahigh Molecular Weight Polyethylene under Artificial Seawater Lubricating Condition. Appl. Surf. Sci..

[18] Ushakova T.M., Starchak E.E., Gostev S.S., Gusarov S.S., Arutyunov I.I., Krasheninnikov V.G., Voskoboynikov A.Z., Novokshonova L.A. (2025). All Polyethylene Compositions Based on Ultrahigh Molecular Weight Polyethylene Synthesized Over Binary Catalyst Including Zirconocenes of Various Designs. Chin. J. Polym. Sci..

[19] Yang H., Hui L., Zhang J., Chen P., Li W. (2017). Effect of Entangled State of Nascent UHMWPE on Structural and Mechanical Properties of HDPE/UHMWPE Blends. J. Appl. Polym. Sci..

[20] Lucas A.D.A., Ambrósio J.D., Otaguro H., Costa L.C., Agnelli J.A.M. (2011). Abrasive Wear of HDPE/UHMWPE Blends. Wear.

[21] Watanabe S., Yamada S., Kasai N., Kida T., Takeshita H., Tokumitsu K. (2024). Effect of Mixing Temperature on the Dispersion and Degradation Behaviors of HDPE/UHMWPE Blends. Int. Polym. Process..

[22] Wang S. (2024). Preparation and Anti-Fouling Modification of Medium-High Strength Polyethylene Fishing Fibers. Master’s Thesis.

[23] Xu L., Huang Y.-F., Xu J.-Z., Ji X., Li Z.-M. (2013). Improved Performance Balance of Polyethylene by Simultaneously Forming Oriented Crystals and Blending Ultrahigh-Molecular-Weight Polyethylene. RSC Adv..

[24] Zhang Q., Lan L., Zheng Z., Liu P., Wu H., Guo S., Lin C., He G. (2022). Constructing Highly Oriented and Condensed Shish-Kebab Crystalline Structure of HDPE/UHMWPE Blends via Intense Stretching process: Achieving High Mechanical Properties and in-Plane Thermal Conductivity. Polymer.

[25] (2024). Plastics—Methods of Exposure to Laboratory Light Sources—Part3: Fluorescent UV Lamps.

[26] (2023). Standard Practice for Operating Fluorescent Ultraviolet(UV) Lamp Apparatus for Exposure of Materials.

[27] Periyasamy A.P., Luoma E., Höhnemann T., Ringger S., Heikkilä P. (2024). Investigate the Processability of Biobased Thermoplastics Used in Nonwoven Fabrics. ACS Polym. Au.

[28] (2022). Man-Made Fibre—Test Method for Tensile Properties of Filament Yarns.

[29] Tarani E., Tara M., Samiotaki C., Zamboulis A., Chrissafis K., Bikiaris D.N. (2024). Preparation and Characterisation of High-Density Polyethylene/Tannic Acid Composites. Polymers.

[30] Jiang T., Mao Z., Qi Y., Wu Y., Zhang J. (2021). The Effect of Two Different UV Absorbers Combined with Antioxidants on UV Resistance of HDPE. Polym. Adv. Technol..

[31] Bao J., Ameyaw S.E., Gao Y., Lu Z., Hu W. (2025). Effect of Ultraviolet Aging on the Structure and Wear Resistance of Ultra-High-Molecular-Weight Polyethylene Ropes. Fibers Polym..

[32] Chen P., Guo P., Guo W., Ding B., Dou H., Zhang X. (2025). Identifying interface evolutions for achieving stable solid-state Li metal batteries. J. Energy Chem..

[33] Liu H.-C., Zhou X.-X., Zhou S.-S., Zou Y., Wang Y.-L., Li X.-Q. (2025). Preparation and Properties Study of Itaconic Acid-based Degradable Epoxy Resin Based on Dynamic Covalent Bonds. Chin. J. Polym. Sci..

[34] Šlouf M., Gajdošová V., Šloufová I., Lukešová M., Michálková D., Müller M.T., Pilař J. (2024). Degradation Processes in Polyolefins with Phenolic Stabilizers Subjected to Ionizing or Non-Ionizing Radiation. Polym. Degrad. Stab..

[35] AlSalem F., Louhichi A., Rastogi S. (2024). Melt Blending of Commercial Linear Polyethylene with Low-Entangled Ultra-High Molecular Weight Polyethylene: From Dispersion Compatibility to Viscoelastic Scaling Laws. Polymer.

[36] Safandowska M., Makarewicz C., Rozanski A., Idczak R. (2023). Diminishment the Gas Permeability of Polyethylene by “Densification” of the Amorphous Regions. Sci. Rep..

[37] Gao J.-W., Chen L., Zhong Y.-S., Xing C.-W., Li Y.-G., Wang Z.-B. (2024). Structural Evolution of Ultra-High Molecular Weight Polyethylene Low-Entangled Films with Reserved Shish Crystals During Hot Stretching. Chin. J. Polym. Sci..

[38] Zaharescu T., Nicula N., Râpă M., Iordoc M., Tsakiris V., Marinescu V.E. (2023). Structural Insights into LDPE/UHMWPE Blends Processed by γ-Irradiation. Polymers.

[39] Shelly D., Lee S.-Y., Park S.-J. (2024). Compatibilization of Ultra-High Molecular Weight Polyethylene (UHMWPE) Fibers and Their Composites for Superior Mechanical Performance: A Concise Review. Compos. Part B Eng..

[40] Ahmed S., Cardinaels R., Abu-Jdayil B., Munam A., Iqbal M.Z. (2025). Toughening Brittle Poly(Ethylene Furanoate) with Linear Low-Density Polyethylene via Interface Modulation Using Reactive Compatibilizers. ACS Omega.

[41] Shirvanimoghaddam K., Balaji K.V., Ahmadi M., Ajdari Nazarloo H., Yadav R., Zabihi O., Egan B., Adetunji P., Naebe M. (2024). Strategies to Resolve Intrinsic Conflicts between Strength and Toughness in Polyethylene Composites. Adv. Ind. Eng. Polym. Res..

[42] Yu J., Zhao X. (2023). Research Progress on the Preparation Method of Anti-Aging Polyolefins. Appl. Chem. Ind..

[43] Study on the Aging Behavior of an Ultra-High Molecular Weight Polyethylene Fiber Barrier Net in a Marine Environment. https://www.mdpi.com/1996-1944/15/16/5599.

[44] Liu J., Li C., Yang Y., Wang W., Guo F., Jing K., Wang L. (2025). Ultra-High Molecular Weight Polyethylene: From Synthesis to Applications. J. Organomet. Chem..

[45] Deng Q., He B., Shen M., Ge J., Du B., Zeng L. (2024). First Evidence of Hindered Amine Light Stabilizers As Abundant, Ubiquitous, Emerging Pollutants in Dust and Air Particles: A New Concern for Human Health. Environ. Sci. Technol..

